# CONUT Score: A New Tool for Predicting Prognosis in Patients with Advanced Thyroid Cancer Treated with TKI

**DOI:** 10.3390/cancers14030724

**Published:** 2022-01-30

**Authors:** Cristina Dalmiglio, Lucia Brilli, Michele Campanile, Cristina Ciuoli, Alessandra Cartocci, Maria Grazia Castagna

**Affiliations:** 1 Department of Medical, Surgical and Neurological Sciences, University of Siena, 53100 Siena, Italy; cristina.dalmiglio@student.unisi.it (C.D.); lucia.brilli@ao-siena.toscana.it (L.B.); michele.campanile@student.unisi.it (M.C.); c.ciuoli@ao-siena.toscana.it (C.C.); 2 Department of Medical Biotechnologies, University of Siena, 53100 Siena, Italy; alessandra.cartocci@dbm.unisi.it

**Keywords:** CONUT score, thyroid cancer, tyrosine kinase inhibitors

## Abstract

**Simple Summary:**

Many studies have shown that an impaired nutritional status correlated with a worse prognosis in cancer patients. The aim of our retrospective study was to evaluate the potential role of baseline Controlling Nutritional Status (CONUT score) in predicting prognosis of advanced thyroid cancer treated with tyrosine kinase inhibitors (TKI). We were able to confirm that baseline CONUT score significantly correlated with progression free survival (PFS) and overall survival (OS) and was the only independent prognostic factor for both outcomes. In particular, a CONUT score ≥3 was associated with a worse PFS and OS. The CONUT score represents a relatively new screening tool that is useful in predicting prognosis in thyroid cancer patients before the beginning of anti-tumoral treatment.

**Abstract:**

(1) Background: The Controlling Nutritional Status (CONUT) score is an immuno-nutritional screening tool based on serum albumin, total cholesterol, and lymphocyte count. The aim of the study was to assess the CONUT score as a potential prognostic factor of response to therapy in patients with advanced thyroid cancer treated with tyrosine kinase inhibitors (TKIs). (2) Methods: We retrospectively evaluated 42 metastatic thyroid cancer patients (54.8% female). The median age at the time of TKI treatment was 69 years. Histological diagnosis was differentiated thyroid cancer in 66.7%, poorly differentiated thyroid cancer in 21.4%, and medullary thyroid cancer in 11.9% of patients. CONUT score was assessed before starting TKI therapy. (3) Results: Progression-free survival (PFS) and overall survival (OS) were significantly influenced by baseline CONUT score. The best CONUT cut-off able to predict the response to treatment was 3. Both PFS and OS were better in patients with CONUT score <3 than in those with CONUT score ≥3 (*p* < 0.0001). CONUT score was the only independent prognostic factor associated with PFS (*p* = 0.021) and OS (*p* = 0.007). (4) Conclusions: CONUT score represents a relatively new screening tool, easily applicable in clinical practice and potentially useful in predicting prognosis in thyroid cancer patients treated with TKIs.

## 1. Introduction

Tyrosine kinase inhibitors (TKIs) are a new class of oncological drugs with activity against receptors of different growth factors and able to inhibit pathways involved in tumor cell proliferation and neoangiogenesis [1]. They have been approved in many tumors including thyroid cancer as they play a crucial therapeutic role when conventional treatments are no longer effective [2]. To date, different TKIs have been approved by the Food and Drug Administration (FDA) and European Medical Agency (EMA) for RAI-refractory differentiated thyroid cancer (sorafenib and lenvatinib) and medullary thyroid cancer (vandetanib and cabozantinib), while dabrafenib/trametinib combination has obtained regulatory approval by the FDA for anaplastic thyroid cancer with a *BRAF* V600 mutation [3,4,5,6,7].

It has been demonstrated that TKIs significantly improved the progression free survival of patients with advanced disease; on the other hand, they are frequently associated with adverse events, which may affect the quality of life or request a permanent drug withdrawal in about 20% of cases [3,4,5,6,7,8]. TKIs are often associated with weight loss, anorexia, fatigue, and gastrointestinal side effects that may contribute to a malnutrition state.

Malnutrition is a common finding in cancer patients with advanced disease. It is associated with a reduction in physical function, it may negatively affect the prognosis and interfere with the anti-cancer treatment [9]. The energy deficit and the loss of lean body mass are related to the reduced food intake and metabolic disorders (increased basal metabolic rate, insulin-resistance, catabolic processes induced by cytokines and inflammatory factors), but anti-cancer therapy itself can also promote malnutrition.

Recent studies have shown the importance of single nutritional indices such as serum albumin in predicting poor outcomes in cancer patients [10,11,12]. Additionally, more complex indices have been developed and validated in order to evaluate the nutritional status [13,14]. The Controlling Nutritional Status (CONUT) score has recently been introduced as a nutritional screening tool [15]. Moreover, it has been recognized as a prognostic factor in patients affected by several chronic or malignant diseases [16,17,18,19,20,21,22,23,24,25,26].

Its utility has been demonstrated in the assessment of the prognosis of patients with end-stage liver disease and acute heart failure [16,17]; it has also been correlated with the disease activity in patients with lupus nephritis [27]. The impact of CONUT score on survival has also been reported in hospitalized elderly people [28] and in patients with hypertension [18] or in peritoneal dialysis [19]. Moreover, the prognostic role of CONUT score has been widely investigated in the context of several types of neoplasms. Many studies have demonstrated how a low CONUT score at the baseline correlates with a better prognosis in patients with small cell lung cancer, gastrointestinal, pancreatic, ovarian, breast, and urological cancers [20,21,22,23,24,25,26]. It has also been shown to be a parameter that correlates with prognosis and response to treatment in oncology [29].

To date, there are no studies in the literature that have investigated the relationship between CONUT score and thyroid cancer.

The aim of our study was to assess the CONUT score as a potential prognostic factor of response to therapy in patients with advanced thyroid cancer (differentiated, medullary, or poorly differentiated thyroid cancer) treated with TKIs.

## 2. Materials and Methods

### 2.1. Study Population

We retrospectively evaluated 71 patients with locally advanced or metastatic thyroid carcinoma treated with at least one TKI at our institution between November 2004 and October 2020.

The data collected included age at diagnosis, gender, histological findings, stage at diagnosis, numbers of anatomical site involved, information on treatment with TKIs (type of TKI, time-lapse between diagnosis and treatment start, duration of treatment, reason for discontinuation), tumor response, and data on last follow-up/death.

Patients without available complete biochemical data and those receiving statin treatment for cholesterol control with a serum total cholesterol <180 mg/dl were excluded from our study.

The final population was made of 42 patients (54.8% female). The mean age at the time of TKI treatment was 67.5 ± 13.8 years (median 69 years, 30–96 years). All patients had metastatic disease. In detail, the number of anatomical sites involved by metastatic disease was one in five patients (11.9%), two in 10 patients (23.8%), three in 18 patients (42.8%), four in four patients (9.5%), and five in five patients (11.9%). Histological diagnosis was differentiated thyroid cancer (DTC) in 28 patients (66.7%), poorly differentiated thyroid cancer (PDTC) in nine patients (21.4%), and medullary thyroid cancer (MTC) in five (11.9%).

Time-lapse between cancer diagnosis and TKI treatment start ranged from 0.06 to 15.4 years (mean 5.4 ± 4.4, median 5.4 years) and ranged from 0 to 14.2 years (mean 3.43 ± 3.5, median 2.8 years) between the appearance of metastases and TKI treatment beginning.

The TKI used as first-line therapy was: lenvatinib in 16 patients (38.1%), sorafenib in 18 patients (42.8%), vandetanib in four patients (9.5%), and motesanib in four patients (9.5%). Ten out of the 42 patients (23.8%) were treated with other TKI.

The median body mass index (BMI) at baseline was 26.5 kg/m^2^ (mean 27.2 ± 6.0 range 18.1–47.0 kg/m^2^). The performance status at baseline, according to the Eastern Cooperative Oncology Group (ECOG) scale, was 0 in 36 patients (85.7%), one in four patients (9.5%), and two in two patients (4.8%).

All data are summarized in Table 1.

### 2.2. Assessments and Definitions

We evaluated in all patients the following anthropometric parameters: weight, height, and body mass index (BMI). The weight (kilograms) and height (meters) were measured using a scale with an altimeter (Seca-Intermed, Milan, Italy). The BMI was calculated as ‘weight over height square’.

At baseline (before starting TKI treatment), fasting venous blood sample was collected for biochemical tests including albumin, total cholesterol, and total lymphocyte count. The concentration of such parameters was measured with standard colorimetric methods using the Cobas c 701/702 analyzer (Roche/Hitachi, Mannheim, Germany). The CONUT score was defined as the sum of the following parameters, as described below:

- for serum albumin levels >3.5, between 3.0 and 3.49, between 2.5 and 2.99 and <2.5 g/dl, 0, 2, 4, and 6 points were assigned, respectively.
- for serum total cholesterol levels >180, between 140 and 179, between 100 and 139 and <100 mg/dl, 0, 1, 2, and 3 points were assigned, respectively.
- for serum total lymphocyte count >1600, between 1200 and 1599, between 800 and 1199 and <800 / mm^3^, 0, 1, 2, and 3 points were assigned, respectively.

Radiological evaluation was performed at baseline (before treatment) and on average every 3–6 months thereafter with computed tomography (CT) scanning with contrast medium or magnetic resonance imaging (MRI).

The time from TKI administration to the first evidence of tumor progression or until death defines the progression free survival (PFS). Tumor progression was documented by CT or MRI examination according to Response Evaluation Criteria in Solid Tumors (RECIST) v 1.1. Best response (BR) was defined as the best response recorded from the start of the treatment until disease progression. The time from the start date of the TKI treatment to the time of death from any cause defines the overall survival (OS).

### 2.3. Statistical Analysis

A preliminary descriptive analysis was performed: quantitative variables were summarized by mean ± standard deviation, median and minimum–maximum range, and qualitative variables by absolute frequencies and percentages. Kruskal test and the post hoc Dunn test were performed to compare the differences of OS and PFS among the three CONUT score ranges (0–2, 3–4, 5–7). Multiple chi-squared test with Bonferroni correction was performed to compare the response (partial response/stable disease and progressive disease) among the three CONUT score levels. ROC curve was performed to obtain a CONUT score cut-off for the most accurate prevision of PFS and OS at 12 months.

The associations with the dichotomous CONUT score (0–2 and 3–7) were evaluated with the chi-squared test or Fisher exact test. The differences between low and high CONUT were evaluated with the *t*-test or the Mann–Whitney test based on the Kolmogorov–Smirnov test for the normality distribution.

Kaplan–Meier and Cox regression analyses were used to assess progression free survival and overall survival. Hazard ratio and their 95% confidence interval (CI) were estimated. Stepwise Cox regression was performed. A *p*-value < 0.05 was considered statistically significant. The analyses were performed with SPSS statistics version 27.

## 3. Results

### 3.1. Baseline CONUT Score (before TKI Treatment)

The median baseline CONUT score was 2 (range 0–7). In detail, it was 0 in 9/42 patients (21.4%), one in 10/42 patients (23.8%), two in 9/42 patients (21.4%), three in 5/42 patients (11.9%), four in 4/42 patients (9.5%), five in 3/42 patients (7.1%), six in 1/42 patients (2.4%), and seven in 1/42 patients (2.4%).

### 3.2. TKI Treatment

The median duration of first TKI therapy was 15.7 months (mean 23.4 ± 19.3 months, range 0.9–76.2 months). At the last follow-up, 9/42 (21.4%) patients were still being treated, 27/42 (64.3%) patients discontinued therapy due to disease progression [20/27 (74.1%)] or other reasons (adverse events, patient decision) [7/27 (25.9%)], while 6/42 (14.3%) patients died.

The median PFS was 12.9 months (mean 16.6 ± 15.1, range 0.9–71.7 months). The best response, evaluable in 40/42 patients (95.2%), was partial response (PR) in 14/40 patients (35%), stable disease (SD) in 21/40 patients (52.5%), and progressive disease (PD) in 5/40 patients (12.5%).

The OS was assessed in a sub-population of 32 patients treated with only one TKI to avoid the potential bias due to those patients treated with more than one TKI. The median OS in this group was 16.8 months (mean 19.1 ± 13.4, range 0.9–56.1 months).

### 3.3. Correlation between Baseline CONUT Score and Response to TKI Treatment

In order to evaluate a possible correlation between nutritional status and the response to first TKI treatment, patients were stratified into three groups according to CONUT score value. We observed that patients with a CONUT score 0–2 (Group A) had a significantly better PFS and OS than patients with a CONUT score 3–4 (Group B) (*p* = 0.001 for PFS and *p* = 0.006 for OS) and with a CONUT score 5–7 (Group C) (*p* = 0.02 for PFS, *p* = 0.002 for OS). Accordingly, we also observed that patients with a CONUT score 0–2 had a significantly better response, according to RECIST criteria, than the other two groups (*p* = 0.002 and *p* = 0.003, respectively). In contrast, PFS, OS, and radiological response were not significantly different between patients with a CONUT score 3–4 (Group B) and 5–6 (Group C) (Table 2).

### 3.4. ROC Analysis for 12 Months—PFS and OS

**Figure 1:**
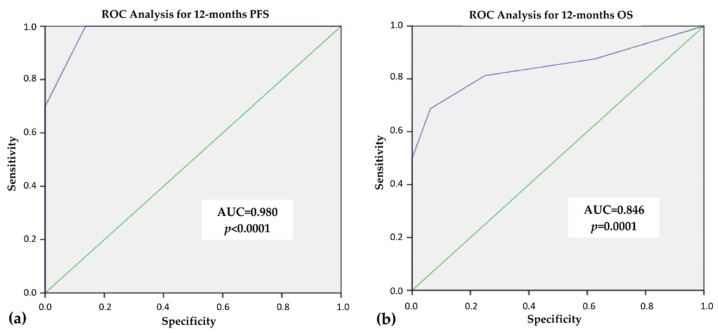
ROC curve analysis for 12-month progression free survival (PFS) (**a**) and 12-month overall survival (OS) (**b**).

By ROC curves analysis, we found that the best CONUT score cut-off able to predict the response to TKI treatment was 3 (Table S1). This cut-off had a specificity of 100% and 93.7% and a sensitivity of 70% and 68.8% for PFS and OS, respectively, with an Area Under the Curve (AUC) of 0.980 for mPFS, (*p* < 0.0001) and of 0.846 for mOS (*p* = 0.0001), as showed in Figure 1.

### 3.5. Clinical-Pathological Features in Thyroid Cancer Patients with CONUT Score <3 (Group 1) and ≥3 (Group 2)

According to the cut-off obtained by ROC analysis, the study population was divided into two groups: Group 1 (CONUT score <3) and Group 2 (CONUT score ≥3). No significant differences between the two groups regarding all clinical-pathological data collected (gender, age at beginning of treatment, time-lapse between cancer diagnosis and treatment, time-lapse between appearance of metastasis and treatment, tumor histotype, numbers of anatomical site involved, rate of bone metastasis, type of TKI, BMI, and ECOG PS) were observed in Group 1 compared to Group 2 (Table S2).

### 3.6. Clinical Outcomes According to CONUT Score

**Figure 2:**
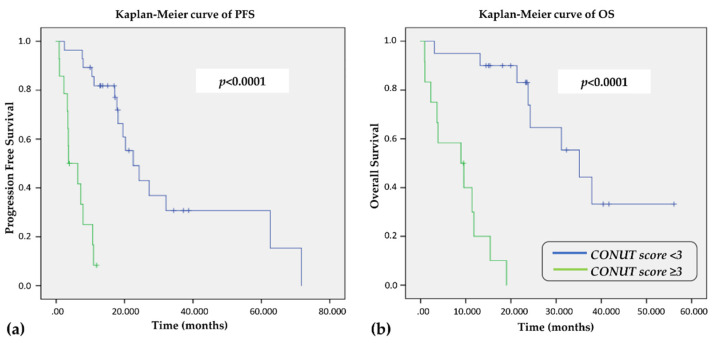
Kaplan–Meier curve for PFS (**a**) and OS (**b**) according to the CONUT cut-off of 3.

Using Kaplan–Meier curves, a significant better PFS was observed in Group 1 patients when compared to Group 2 (22.5 vs. 5.0 months, *p* < 0.0001, Figure 2a). Similarly, the OS was longer in Group 1 than Group 2 (35.1 vs. 9.2 months, *p* < 0.0001, Figure 2b). All but one patient died for thyroid disease, confirming a better disease specific survival in Group 1 than in Group 2.

We also performed a sub-analysis of PFS and OS according to the histology. Prognostic power of the CONUT score was still valid in DTC patients (OS: HR 1609, 95% CI 1.232–2.12, *p* < 0.0001; PFS: HR 1.505, 95% CI 1.186–1.908, *p* = 0.001), but not in MTC and PDTC patients, probably due to the small cohort of patients in these two groups.

### 3.7. Univariate and Multivariate Analysis for PFS and OS

At univariate Cox-regression analysis, serum albumin level [HR 0.408 (95% CI 0.168–0.991), *p* = 0.048], total lymphocyte count [HR 0.999 (95% CI 0.999–1.00), *p* = 0.035], and baseline CONUT score [HR 12.211 (95% CI 4.084–36.512), *p* < 0.0001] were prognostic factors for PFS as well as serum albumin level [HR 0.224 (95% CI 0.083–0.604), *p* = 0.003], serum total cholesterol [HR 0.982 (95% CI 0.969–0.995), *p* = 0.007], and CONUT score [HR 23.551 (95% CI 4.949–112.060), *p* < 0.0001] were prognostic factors for OS. According to stepwise Cox-regression analysis, the CONUT score was the only independent prognostic factor associated with PFS (*p* < 0.0001) and OS (*p* < 0.0001), whose HRs were confirmed to be 12.211 (95% CI 4.084–36.512) for PFS and 23.551 (95% CI 4.949–112.060) for OS.

## 4. Discussion

Recently, the prognostic value of nutritional status and inflammation has been de-bated in cancer patients [30,31]. Many studies have demonstrated that an impaired nutritional status and an increased inflammatory response are related to a worse prognosis in patients with different types of human cancer [32,33]. Different biochemical parameters have been proposed to evaluate the immune-nutritional status such as blood neutrophil, lymphocyte, monocyte, platelet count, neutrophil–lymphocyte ratio (NLR), lymphocyte–monocyte ratio (LMR), and platelet–lymphocyte ratio (PLR) [34,35,36]. Immune-nutritional scores such as the Prognostic Nutritional Index (PNI) and the Controlling Nutritional Status (CONUT) have also recently been evaluated in human cancer [20,21,22,23,24,25,26,37]. The latter has recently been implemented as a tool of nutritional screening, emerging as an independent prognostic factor for OS in several types of solid tumors [38] as well as in hematological cancer [39,40].

To our knowledge, our study is the first to investigate the potential role of the baseline CONUT score as a prognostic factor for PFS and OS in patients with advanced thyroid cancer (differentiated, medullary, or poorly differentiated thyroid cancer) treated with TKIs. In recent years, TKIs have become a pivotal therapy in many types of cancer [2]. These drugs are scheduled for chronic administration and are often associated with systemic side effects favoring malnutrition (i.e., diarrhea, nausea, mucositis), if not properly managed [8].

Our cohort of patients showed an overall baseline good nutritional status, with a CONUT score suggestive of moderate malnutrition only in 5/42 (11.9%) patients, while none had severe grade of malnutrition (highest CONUT score recorded equal to 7). Nevertheless, a significant correlation between immuno-nutritional status and clinical outcome was observed. Using ROC curve analysis, we found that a cut-off of 3 was able to predict the response to treatment with 100% of specificity and 70% of sensitivity for PFS (AUC 0.980, *p* < 0.0001), and 93.7% of specificity and 68.8% of sensitivity for OS (AUC 0.840, *p* = 0.001). It is important to underline that the two groups of patients (CONUT score <3 and ≥ 3) were similar for clinical and pathological features that could have an impact on the clinical outcome. Nevertheless, the number of patients that took the full dosage did not differ between the two groups at the beginning of TKI treatment as well as at the last follow-up. Since TKI dosage is mostly related to the adverse events, we can assume that the tolerance to therapy was also similar in the two groups of patients.

The optimal cut-off of the CONUT score varied across different studies [38]; in our study, it was higher compared to that found in patients with small cell lung cancer [20], but lower compared to that calculated in other cancer studies [21,22,23,29]. This difference is probably due to the different nutritional status and pathogenic mechanisms underlying the various types of cancer.

Multiple potential mechanisms underlying the relationship between parameters included in the CONUT score and cancer prognosis have been widely analyzed. The serum albumin reflects both the nutritional and the inflammatory status and is considered as a prognostic factor in several cancers [10,11,12]. It has been postulated that pro-inflammatory cytokines such as interleukin 6 (IL-6) and tumor necrosis factor alpha (TNF-α), which modulate albumin hepatic synthesis, are associated with lower serum concentrations of this protein [41,42]. TNF-α induces gluconeogenesis and the lipid and muscle protein catabolism. It also stimulates the production of reactive oxygen species (ROS) in tissues, leading to the activation of ubiquitin-proteasome pathway and inducing muscle protein catabolism [43]. Moreover, in preclinical studies, it has been demonstrated that TNF-α and interleukin 1 (IL-1) penetrate the hematoencephalic barrier, causing an anorexigenic effect [44]. The protein deficit causes a downregulation of hormonal and antibody synthesis, leading to a suppression in B-cell differentiation and T-cell activation [45].

There are many assumptions about the relationship between serum cholesterol and prognosis in cancer [46,47,48]. Okuyama et al. [49] and many other authors have demonstrated a correlation between hypocholesterolemia and worse prognosis in non-small cell lung cancer, gastric cancer, and localized renal cell carcinoma [50,51,52]. It seems that tumoral cells can determine a reduction in serum cholesterol through many mechanisms. such as an increased uptake into the cells caused by the exposition of low-density lipoprotein (LDL)-cholesterol receptors on the cell membrane [53].

The lymphocyte count is widely used in prognostic scores since the immune response against cancer largely depends on lymphocytes. CD4+ and CD8+ lymphocytes are implied in the prevention of neoplastic proliferation and invasion, CD8+ T cell counts are consistently associated with better survival in many types of cancer [54,55,56].

Interestingly, the CONUT score cut-off found in our study was the only independent parameter associated with PFS (HR 12.2, *p* < 0.0001) and OS (HR 23.55, *p* < 0.0001) by multivariate stepwise Cox-regression analysis, suggesting a possible role of nutritional status in the clinical outcome of thyroid cancer patients with advanced disease treated with anticancer therapy.

The study has a few limitations such as the presence of multiple histotypes of thyroid cancer, the use of different TKIs, the small sample size, and the retrospective design of the study.

However, the study has several strengths such as standardized management in the same institution with detailed information regarding diagnosis, treatment, and follow-up. Finally, to our knowledge, this is the first study to have evaluated the role of CONUT score as an immuno-nutritional tool in the clinical outcome of advanced thyroid carcinoma treated with TKIs.

## 5. Conclusions

The CONUT score represents a relatively new screening tool, easily applicable in clinical practice with a possible prognostic role in the management of patients with advanced thyroid cancer treated with TKIs.

Based on this evidence, the nutritional status should be taken into account in meta-static thyroid cancer patients, since the efficacy of anticancer treatments could potentially be impaired by malnutrition. The improvement in the nutritional status of cancer patients before starting and/or during TKI treatment could be one of the strategies to obtain the maximum efficacy of anticancer therapy. This study shows promising and innovative results, opening the way to future prospective studies on a larger sample size to strengthen the correlation between the nutritional status, evaluated at baseline and during cancer treatment, and the response to TKI treatment in patients with advanced thyroid cancer.

## Figures and Tables

**Table 1:** Baseline clinical-pathological features in the whole population and according to histological diagnosis.

	All Patients	DTC	PDTC	MTC
	(*n* = 42)	(*n* = 28)	(*n* = 9)	(*n* = 5)
Gender *n* (%)				
F	23 (54.8%)	18 (64.3%)	3 (33.3%)	2 (40%)
M	19 (45.2%)	10 (35.7%)	6 (66.7%)	3 (60%)
Age at the time of TKI treatment (years) Median (range)	69 (30–9)	71.6 (43–87)	67.2 (51.2–96.2)	51.4 (30–80)
Time-lapse between cancer diagnosis and TKI treatment (years) Median (range)	5.4 (0.06–15.4)	5.71 (0.09–15.4)	1.96 (0.06–12)	7.7 (1.1–9.9)
Time-lapse between appearance of metastases and TKI treatment (years) Median (range)	2.8 (0–14.2)	3.2 (0.03–14.2)	1.2 (0–12)	1.1 (0.6–4.9)
Numbers of anatomical site involved *n* (%)				
1	5 (11.9%)	4 (14.3%)	1 (11.1%)	0 (0%)
2	10 (23.8%)	8 (28.6%)	1 (11.1%)	1 (20%)
≥3	27 (64.3%)	16 (57.1%)	7 (77.8%)	4 (80%)
Patients with bone metastasis *n* (%)	15 (35.7%)	9 (32.1%)	3 (33.3%)	3 (60%)
Type of first-line TKI *n* (%)				
Lenvatinib	16 (38.1%)	10 (35.7%)	6 (66.7%)	0 (0%)
Sorafenib	18 (42.8%)	14 (50%)	3 (33.3%)	1 (20%)
Vandetanib	4 (9.5%)	2 (7.1%)	0 (0%)	2 (40%)
Motesanib	4 (9.5%)	2 (7.1%)	0 (0%)	2 (40%)
BMI (kg/m^2^) Median (range)	26.5 (18.1–47)	26.9 (18.7–44.1)	24.6 (18–46.9)	24.8 (22.7–34.4)
ECOG PS *n* (%) ^1^				
0	31 (86.1%)	20 (83.33%)	7 (87.5%)	4 (100%)
1	3 (8.3%)	2 (8.33%)	1 (12.5%)	0 (0%)
2	2 (5.6%)	2 (8.33%)	0 (0%)	0 (0%)

^1^ ECOG PS was evaluated in 36 patients.

**Table 2:** Response to TKIs according to baseline CONUT score (Group A score 0–2, Group B score 3–4, Group C score 5–7).

	Patients (*n*; %)	BR1 (*n*; %)	PFS ^1^ Median (Months) Range	OS ^2^ Median (Months) Range
Group A (CONUT score 0–2)	28 (66.7%)	PR 10/27 (37%)	17.9 (2.4–71.7)	23.5 (3.1–56.1)
		SD 17/27 (63%)		
		PD 0/27 (0%)		
Group B (CONUT score 3–4)	9 (21.4%)	PR 3/8 (37.5%)	3.6 (0.9–11.8)	8.9 (0.9–19.0)
		SD 2/8 (25%)		
		PD 3/8 (37.5%)		
Group C (CONUT score 5–7)	5 (11.9%)	PR 1/5 (20%)	7.9 (2.2–10.9)	9.6 (2.2–15.4)
		SD 2/5 (40%)		
		PD 2/5 (40%)		
		p A vs. B = 0.002	p A vs. B = 0.001	p A vs. B = 0.0006
		p A vs. C = 0.003	p A vs. C = 0.0224	p A vs. C = 0.0028
		p B vs. C = 0.759	p B vs. C = 0.737	p B vs. C = 0.923

^1^ BR and PFS were assessed in 40 patients. ^2^ OS was evaluated in 32 patients treated with only one TKI.

## Data Availability

Data are contained within the article.

## Supplementary Materials

The following supporting information can be downloaded at: https://www.mdpi.com/article/10.3390/cancers14030724/s1, Table S1: CONUT score cut-offs according to ROC analysis for Progression Free Survival (a) and Overall Survival (b); Table S2: Clinical-pathological features in thyroid cancer patients with CONUT score <3 (Group 1) and ≥3 (Group 2).
