# Supplementary material for: CONUT Score: A New Tool for Predicting Prognosis in Patients with Advanced Thyroid Cancer Treated with TKI
Source: Cancers (Basel). 2022 Jan 30;14(3):724. doi: 10.3390/cancers14030724 (PMC8833681; doi:10.3390/cancers14030724)
Supplement: Supplementary file 1 [file cancers-14-00724-s001.zip › cancers-1530274-supplementary.pdf]

Supplementary Table S1. CONUT score cut-offs according to ROC analysis for Progression Free Survival (a) and Overall Survival (b).

**(a) Progression Free Survival**			
CONUT score cut-off	*HR*	*95% CI*	*p value*
1	3.499	1.042-11.74	0.046
2	3.895	1.707-8.888	0.001
**3**	**12.21**	**4.084-36.51**	**<0.0001**
4	4.322	1.612-11.58	0.004
5	4.304	1.483-12.49	0.007
6	4.318	0.9697-19.23	0.054
**(b) Overall Survival**			
CONUT score cut-off	*HR*	*95% CI*	*p value*
1	9.68	1.287-72.83	0.027
2	9.584	2.757-33.32	<0.0001
**3**	**23.55**	**4.949-112.1**	**<0.0001**
4	11.85	3.372-41.63	0.001
5	5.966	1.835-19.4	0.003
6	3.993	0.8692-18.34	0.075

Supplementary Table S2. Clinical-pathological features in thyroid cancer patients with CONUT score <3 (Group 1) and ≥3 (Group 2).

	All Patients (n=42)	Group 1 CONUT < 3 (n=28)	Group 2 CONUT ≥ 3 (n=14)	p value
Gender n (%)				
F	23 (54.8%)	17 (60.7%)	6 (42.8%)	0.27
M	19 (45.2%)	11 (39.3%)	8 (57.2%)	
Age at the time of TKI treatment (yrs) Median (range)	69 (30-99)	71 (30-96)	66 (54-86)	0.78
Time-lapse between cancer diagnosis and TKI treatment (yrs) Median (range)	5.4 (0.06-15.4)	5.9 (0.06-15.4)	3.2 (0.09-13.9)	0.17
Time-lapse between appearance of metastases and TKI treatment (yrs) Median (range)	2.8 (0-14.2)	3.2 (0.05-12.1)	2.3 (0-14.2)	0.37
Histology n (%)				
DTC	28 (66.7%)	19 (67.8%)	9 (64.3%)	0.63
MTC	5 (11.9%)	4 (14.3%)	1 (7.1%)	
PDTC	9 (21.4%)	5 (17.9%)	4 (28.6%)	
Numbers of anatomical site involved n (%)				
1	5 (11.9%)	4 (14.3%)	1 (7.1%)	0.92
2	10 (23.8%)	7 (25%)	3 (21.4%)	
≥3	27 (64.3%)	17 (60.7%)	18 (71.4%)	
Patients with bone metastasis n (%)	15 (35.7%)	11 (39.3%)	4 (28.6%)	0.49
Type of first-line TKI n (%)	16 (38.1%)	11 (39.3%)	5 (35.7%)	0.88
Lenvatinib	18 (42.8%)	12 (42.8%)	6 (42.8%)	

Sorafenib	4 (9.5%)	3 (10.5%)	1 (7.1%)	
Vandetanib	4 (9.5%)	2 (5.3%)	2 (14.3%)	
Motesanib				
BMI (kg/m²)				0.26
Median (range)	26.5 (18.1-47)	27.5 (18.7-46.9)	25.0 (18.1-31.1)	
ECOG PS n (%)[1]				0.32
0	31 (86.1%)	23 (92%)	8 (72.7%)	
1	3 (8.3%)	2 (8%)	1 (9.1%)	
2	2 (5.6%)	0 (0%)	2 (18.2)	

[1] ECOGS PS was evaluated in 36 patients.
